# Impact of the Enhanced Recovery After Surgery Protocol on the Perioperative Outcomes of Robot-Assisted Radical Cystectomy

**DOI:** 10.3390/jcm14093082

**Published:** 2025-04-29

**Authors:** Kosuke Kitamura, Yuto Miyoshi, Takeshi Ieda, Toshiyuki China, Fumitaka Shimizu, Shigeo Horie, Satoru Muto

**Affiliations:** 1 Department of Urology, Juntendo University Nerima Hospital, 3-1-10 Takanodai, Nerima-ku, Tokyo 177-8521, Japan; kkitamu@juntendo.ac.jp (K.K.); y-miyoshi@juntendo.ac.jp (Y.M.); 2 Department of Urology, Juntendo University, Graduate School of Medicine, Tokyo 113-8421, Japan; tieda@juntendo.ac.jp (T.I.); tchina@juntendo.ac.jp (T.C.); f_simizu@juntendo.ac.jp (F.S.); shrie@juntendo.ac.jp (S.H.)

**Keywords:** urinary bladder neoplasms, cystectomy, enhanced recovery after surgery, robotic surgical procedures, postoperative complications

## Abstract

**Objectives**: The enhanced recovery after surgery (ERAS) protocol is a coordinated approach aimed at providing the best evidence-based perioperative care. This study examined whether combining robot-assisted radical cystectomy (RARC) with the ERAS protocol could reduce postoperative complications and hospital length of stay (LOS). We also assessed the impact of high and low adherence to the ERAS protocol on oncological outcomes. **Methods**: Eighty patients who underwent RARC with urinary diversion at Juntendo University Hospital and Juntendo University Nerima Hospital between April 2014 and December 2021 were included. The ERAS protocol consisted of 15 items, and the achievement rate for each item was assessed. We evaluated the effects of adherence on complications and hospital LOS, as well as the relationship between ERAS implementation and postoperative oncological prognoses. **Results**: Patients were divided into high-adherence (n = 39) and low-adherence (n = 41) groups based on adherence to 12 or more ERAS items. Patient demographics, including age, sex, and clinical stage, were statistically similar. The high-adherence group had a significantly shorter postoperative hospital LOS (19 days vs. 24 days; *p* = 0.013) and fewer complications (*p* = 0.015) compared to the low-adherence group. Furthermore, the high-adherence group exhibited a significantly improved overall survival rate (*p* = 0.029), while no significant difference was found in progression-free survival (*p* = 0.125). **Conclusions**: Integrating the ERAS protocol with RARC can reduce postoperative complications and hospital LOS. High adherence to the ERAS protocol is associated with improved prognoses and outcomes compared to low adherence.

## 1. Introduction

Radical cystectomy and pelvic lymph node dissection with urinary diversion are standard treatments for muscle-invasive bladder cancer. However, robot-assisted radical cystectomy (RARC) has emerged as a minimally invasive procedure with acceptable oncological efficacy and shorter hospital length of stay (LOS) [1]. Given the high rates of perioperative complications associated with radical cystectomy and urinary diversion, the use of the enhanced recovery after surgery (ERAS) protocol has become widespread, demonstrating its utility in reducing complications [2]. The ERAS protocol aims to provide evidence-based perioperative care through multimodal, interdisciplinary pathways that facilitate early recovery, minimize hospital LOS, and reduce complication rates [3]. While several factors influence oncological outcomes during the perioperative period, the long-term benefits of the ERAS protocol for these outcomes are yet to be comprehensively elucidated [4]. This study investigates whether combining RARC with the ERAS protocol can reduce complications and hospital LOS while improving oncological prognoses. We also examine the role of adherence to ERAS items in the oncological outcomes of RARC.

## 2. Materials and Methods

This study included patients who underwent RARC with urinary diversion at Juntendo University Hospital and Juntendo University Nerima Hospital between April 2014 and March 2022. Fifteen ERAS items were developed based on guidelines from the ERAS Society [3]. The preoperative ERAS protocol included counseling on urostomy care/self-catheterization, optimization of medication use and nutrition, prevention of thromboembolism, preoperative carbohydrate loading without prolonged fasting, and avoidance of mechanical bowel preparation and long-acting benzodiazepines. Certified nurses provided preoperative counseling and education regarding the urinary diversion procedure and postoperative management. Guidance from nurses and pharmacists prior to admission helped optimize medication use and nutrition. Preoperative carbohydrate loading was defined as consuming 1000 mL of a 2.5% carbohydrate beverage (OS-1; Otsuka Pharmaceutical Factory, Tokushima, Japan) 1–2 h before surgery. The ERAS protocol for anesthesia recommended thoracic epidural use (T9–T11), minimal opioid use (using short-acting fentanyl if necessary), goal-directed fluid therapy (GDFT), and hypothermia prevention [5]. Patients were fitted with a radial artery catheter and connected to an advanced hemodynamic monitor, with GDFT management based on arterial waveform analysis. Postoperatively, nasogastric intubation was removed to facilitate enteral feeding within the first 24 h, and early mobilization was encouraged. To prevent postoperative paralytic ileus, Daikenchuto, a traditional Japanese herbal medicine, was recommended [6]. The achievement rate for each of the 15 ERAS items was examined (Table 1). The cut-off value for ERAS adherence was calculated using receiver operating characteristic (ROC) curves, and comparisons were made by dividing patients into high- and low-adherence groups. The effects of adherence on complications and hospital LOS were assessed, along with whether ERAS implementation was linked to postoperative adjuvant chemotherapy use and oncological outcomes. This study adhered to the ethical guidelines of the Declaration of Helsinki and received approval from the institutional human research committee. Informed consent was obtained from all participants, and this study was approved by the Institutional Review Board of Juntendo University Graduate School of Medicine (no. 16-237).

### Statistical Analysis

Statistical analyses were performed using JMP software (version 11.0; SAS Institute Inc., Cary, NC, USA). Categorical variables were compared using the t-test, Mann–Whitney U test, and Pearson’s chi-square test. Logistic regression was employed for univariate and multivariate analyses. Kaplan–Meier plots were used to analyze progression-free survival, with differences assessed using the log-rank test. Univariate and multivariate analyses utilized multiple regression and the Cox proportional hazards model. Statistical significance was defined as *p* < 0.05.

## 3. Results

A total of 80 patients who underwent RARC were included. The cut-off value for adherence to the ERAS protocol was determined to be 12 items based on the ROC curve analysis. Patients adhering to 12 or more ERAS items were classified as high-adherence (n = 39), while those adhering to fewer than 12 were classified as low-adherence (n = 41). The characteristics of the patients in each group are presented in Table 2, showing no significant differences in age, body mass index, sex, Charlson comorbidity index, neoadjuvant chemotherapy use, or clinical stage. Table 1 lists the specific ERAS protocol items. Among the high-adherence group, significantly more patients completed all six preoperative items compared to the low-adherence group (61% vs. 10%; *p* < 0.001). Table 3 outlines the perioperative characteristics, operative times, urinary diversion types, pathological outcomes, hospital LOS, and complications for both groups. The high-adherence group experienced significantly shorter operative times (519 min vs. 375 min; *p* < 0.001) and hospital LOS (24 days vs. 19 days; *p* = 0.013) compared to the low-adherence group. Intracorporeal urinary diversions were more prevalent in the high-adherence group. The high-adherence group also had a better complication rate and a lower incidence of serious complications, with an incidence of 16% in the low-adherence group compared with 3% in the high-adherence group (*p* = 0.015). Among complications, there was a differential incidence of postoperative ileus, which was significantly lower in the high-adherence group (14% vs 5%; *p* = 0.043).

**Figure 1:**
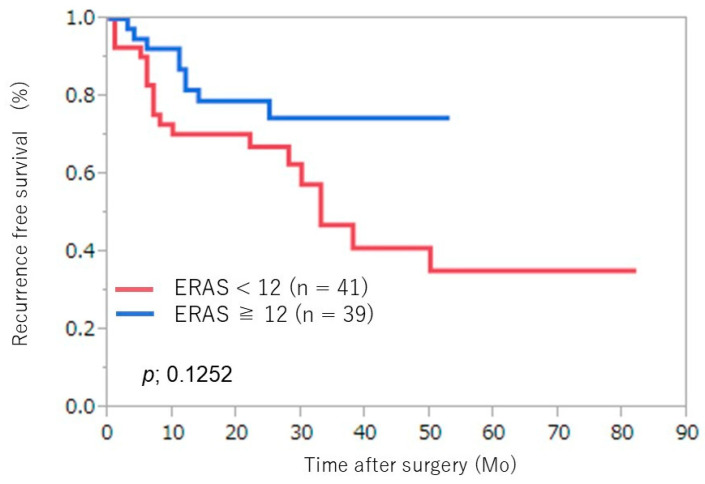
Kaplan–Meier survival curves for recurrence-free survival of the two groups. ERAS, enhanced recovery after surgery. Mo: month.

**Figure 2:**
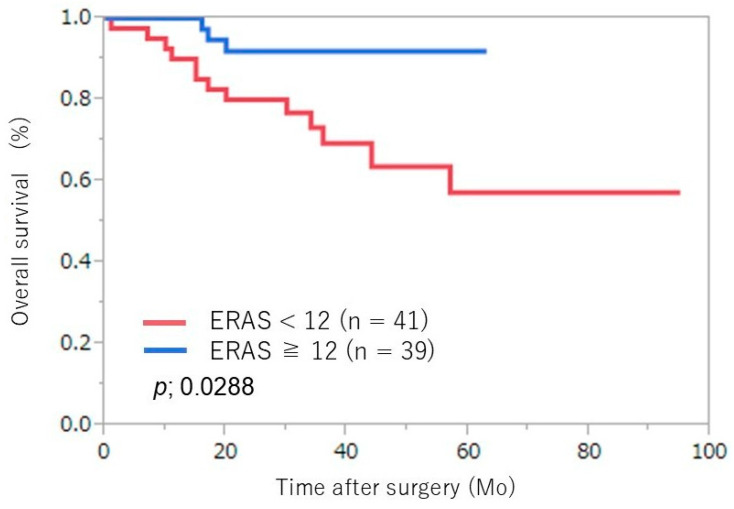
Kaplan–Meier survival curves for overall survival of the two groups. ERAS, enhanced recovery after surgery. Mo: month.

No significant differences in estimated blood loss or pathological outcomes were noted between the groups. Adjuvant treatment consisted of platinum-based chemotherapy, with no significant difference between the two groups (9% vs. 6%; *p* = 0.8734). There were no significant differences in prognosis-related preoperative status, postoperative pathological stage, or adjuvant treatment between groups. An evaluation of the ERAS protocol’s effects on oncological outcomes showed no difference in recurrence-free survival (*p* = 0.125) (Figure 1); however, overall survival was prolonged in the high-adherence group (*p* = 0.029) (Figure 2).

## 4. Discussion

Despite advancements in multimodal treatments for bladder cancer, including radical cystectomy and chemotherapy, surgical invasiveness remains a concern. Therefore, RARC combined with the ERAS protocol aims to mitigate complications and reduce hospital LOS [1,7]. During the preoperative phase, the ERAS protocol encompasses counseling and education on urostomy care and self-catheterization, optimizing medication and nutrition, preoperative carbohydrate loading, avoiding mechanical bowel preparation, using short-acting agents in preanesthetic medication, minimizing fasting periods, and preventing thromboembolism. Based on ERAS guidelines, three easily reportable best-practice measures that are commonly associated with improved outcomes and a compliance rate of 99% were included in this analysis: avoidance of bowel preparation [8], prevention of thromboembolism [9], and avoidance of long-acting benzodiazepines in preanesthetic medications [10]. Optimization begins in the clinical setting with patient education, which is crucial for postoperative recovery. Counseling on urostomy care and nutrition, as well as medication management, contributes to the preoperative optimization of patient status. Education should extend beyond informed consent to encompass radical cystectomy and associated procedures. Patients receiving an ileal conduit for disease management must learn to manage an ostomy, which can significantly alter body image and provoke psychological changes. Effective ostomy management involves mastering daily care procedures and manual skills. Preoperative education programs that adopt an interactive approach can alleviate anxiety and enhance preparation for patients with bladder cancer, thereby significantly reducing hospital LOS [11]. Optimization of medication, nutrition, and physical conditioning can also improve recovery rates [3,12]. A retrospective cohort analysis indicated that exercise, along with the cessation of smoking and drug and alcohol abuse, are vital factors in preventing complications after cystectomy [13]. Preoperative carbohydrate loading using clear liquids with electrolytes and carbohydrates helps mitigate thirst and maintain lean body mass and muscle strength during surgery, consequently shortening recovery time [14,15]. Recommendations for preoperative carbohydrate loading stem from colorectal surgery studies that link carbohydrate intake to reduced insulin resistance and preservation of lean body mass [16]. Carbohydrate solutions exceeding 10% concentration have been shown to positively influence insulin resistance [17]. However, we consider that a 2.5% carbohydrate oral rehydration solution may provide slight improvements in insulin action and be suitable for preventing preoperative dehydration [18,19]. The ERAS protocol advocates for thoracic intraoperative epidural analgesia (T9–T11) to prevent hypothermia and facilitate goal-directed fluid therapy (GDFT). Intraoperative epidural analgesia reduces opioid use and may promote early enteral feeding and mobility [3,14]. Maintaining normothermia during surgery is strongly recommended in various ERAS guidelines as a key aspect of perioperative management and is linked to the prevention of postoperative ileus [3,20]. Fluid overload can result in interstitial edema and local inflammation, negatively impacting tissue healing, while hypovolemia may cause vasoconstriction and insufficient perfusion, leading to organ dysfunction. GDFT is crucial for achieving optimal fluid balance and avoiding complications [21]. The ERAS protocol includes the removal of nasogastric intubation post-surgery, opioid-sparing postoperative analgesia, prevention of postoperative paralytic ileus and nausea/vomiting, initiation of enteral feeding within 24 h postoperatively, and promotion of early mobilization. ERAS guidelines indicate that nasogastric tubes can be removed shortly after extubation in the recovery unit [3]. Optimized postoperative analgesia involves a multimodal, opioid-sparing approach to enhance recovery without adversely affecting postoperative ileus [21]. Daikenchuto (TJ-100), a traditional Japanese herbal medicine, is employed in Japan for the prevention and treatment of postoperative ileus [22]. Multimodal antiemetic prophylaxis is advised for high-risk patients to mitigate postoperative nausea and vomiting [21]. Early mobilization and oral intake after surgery can effectively reduce complications, as well as the time to first flatus and bowel movement [3]. Studies have shown that adherence to the ERAS protocol correlates with fewer complications and a more comprehensive application of protocol items [23]. This study set the ERAS protocol adherence cutoff at 12 out of 15 (80%), similar to findings suggesting that adopting over 15 out of 21 (75%) components could reduce complications [23]. Schiavina et al. reported that patients undergoing RARC were more likely to adhere to the ERAS protocol and experience quicker recovery [24]. All components of the ERAS protocol are reported to be synergistic [25], resulting in higher completion rates of preoperative items among patients with high adherence.

While this study suggests that high adherence to the ERAS protocol is associated with better oncological outcomes, it is important to acknowledge that this association may not be solely attributable to ERAS compliance. Other factors, such as the surgeon’s experience, institutional variations, and patient characteristics, may also contribute to these findings. To more accurately determine the impact of ERAS adherence on oncological outcomes, future studies should employ multivariate analysis or propensity score matching, incorporating factors such as treatment era and clinical stage. Additionally, evaluating the consistency of ERAS effects across different time periods would help confirm whether improvements in outcomes are truly linked to ERAS adherence rather than advancements in surgical techniques or perioperative care. The implementation of the ERAS protocol in cancer surgery can enhance the outcomes of subsequent therapeutic interventions by facilitating early postoperative recovery. Furthermore, compared to low adherence, high adherence to the ERAS protocol is associated with better outcomes [26]. Cancer cell growth is closely linked to tumor aggression towards the patient’s immune system, and surgical procedures may induce a stress response, reducing the body’s natural defenses and promoting tumor growth. ERAS may reduce inflammation during surgery, reduce patient stress, and facilitate the postoperative recovery process, and it is speculated that these effects may collectively contribute to better cancer treatment outcomes [27]. Integrating the ERAS protocol into perioperative management may minimize the inflammatory response to surgery, potentially impacting tumor spread. Additionally, cancer surgeries utilizing the ERAS protocol are more likely to initiate and complete postoperative adjuvant chemotherapy on schedule, reducing surgically induced stress, a significant factor that can promote cancer growth [4]. Although this study did not assess differences in postoperative pathology results and the initiation of adjuvant chemotherapy based on ERAS protocol adherence, it found no difference in adherence concerning progression-free survival. However, the high-adherence group did experience longer overall survival compared to the low-adherence group. While ERAS protocols do not directly affect cancer recurrence or progression, they may be prognostically relevant by minimizing postoperative complications and fostering early recovery. The reduction of postoperative complications and stress through the ERAS protocol may contribute to improved long-term outcomes. Our study has limitations, including a small sample size from only two institutions and comparisons in terms of adherence rates, which may introduce selection bias and unmeasured confounding factors. Notably, differences in the type of urinary diversion were observed between groups, with more intracorporeal urinary diversions performed in the high-adherence group, potentially impacting postoperative outcomes. Although the overall complication rates for intracorporeal and extracorporeal urinary diversion procedures are comparable, intracorporeal methods tend to reduce blood loss and transfusion requirements [28]. Previous randomized controlled trials have shown that RARC with intracorporeal urinary diversion reduces transfusion rates and complications while achieving comparable oncological outcomes [29]. While we considered that differences in urinary diversion may have influenced outcomes, no direct association was established.

The treatment described in this study differs from current recommendations for muscle-invasive bladder cancer, as no patients received adjuvant nivolumab. A phase 3, multicenter, double-blind, randomized controlled trial involving patients with high-risk muscle-invasive urothelial carcinoma post-radical surgery found that disease-free survival with adjuvant nivolumab significantly exceeded that of the placebo [30]. Adjuvant therapy can influence postoperative outcomes; thus, the prognostic impact of high adherence to the ERAS protocol may also affect these outcomes. Both scheduled adjuvant therapy and high adherence to the ERAS protocol likely confer positive prognostic benefits for cancer surgery patients. Although ERAS protocol implementation varies across institutions, this study suggests that contemporary perioperative care measures are effective in improving outcomes. The ERAS protocol was easily implemented in this study, and future multicenter prospective studies are encouraged.

## 5. Conclusions

Clinical evidence indicates that the ERAS protocol enhances short-term outcomes following radical cystectomy, resulting in fewer complications and shorter hospital stays. Our findings demonstrate that combining the ERAS protocol with RARC can effectively reduce complications and hospital LOS. Moreover, high adherence to the ERAS protocol is linked to improved prognoses and outcomes compared to low adherence.

## Figures and Tables

**Table 1:** Items included in the ERAS protocol and their achievement rates.

ERAS Item	Achievement Rate
Training regarding performing urostomy care/self-catheterization	70%
Explanation of medication use and nutritional management	63%
Avoidance of oral bowel preparation	99%
Avoidance of preoperative long-acting sedatives	99%
Carbohydrate loading	48%
Long-term use of prophylaxis to avoid thromboembolism	99%
Goal-directed perioperative fluid management	44%
Prevention of intraoperative hypothermia	79%
Use of epidural analgesia	95%
Avoidance of nasogastric intubation	91%
Pain management with opioid-sparing analgesics	86%
Prevention of paralytic ileus	89%
Prevention of nausea and vomiting	76%
Early ambulation within 24 h postoperatively	60%
Early return to an oral diet	65%

ERAS, enhanced recovery after surgery.

**Table 2:** Comparison of the preoperative characteristics of the two groups.

	Adherence to <12 ERAS Items (n = 41)	Adherence to ≥12 ERAS Items (n = 39)	*p*
Median age, years (IQR)	70 (58–74)	68 (65–73)	0.2953
Median BMI, kg/m^2^ (IQR)	23.9 (21.1–26.0)	23.9 (22.3–25.0)	0.9102
Sex, no. (%)			0.5526
Male	35 (44)	35 (44)	
Female	6 (7)	4 (5)	
Charlson comorbidity index, no. (%)			0.5908
0	17 (21)	22 (28)	
1–2	23 (29)	16 (20)	
≥3	1 (1)	1 (1)	
Clinical stage, no. (%)			0.2310
cT1–2	31 (39)	31 (39)	
cT3–4	10 (12)	8 (10)	
Neoadjuvant chemotherapy, no. (%)			0.9105
None	7 (9)	8 (10)	
1	8 (10)	7 (9)	
≥2	26 (32)	24 (30)	

**Table 3:** Comparison of the perioperative characteristics of the two groups.

	Adherence to <12 ERAS Items (n = 41)	Adherence to ≥12 ERAS Items (n = 39)	*p*
**Operative time (min), median (IQR)**	519 (453–613)	375 (321–438)	0.0001 *
**Blood loss (mL), median (IQR)**	310 (190–550)	232 (155–466)	0.1569
**Urinary diversion type, no. (%)**			0.0013 *
ECUD IC	8 (10)	0 (0)	
ECUD NB	15 (19)	6 (8)	
ICUD IC	18 (22)	30 (37)	
ICUD NB	0 (0)	3 (4)	
**Hospital LOS (day), median (IQR)**	24 (19–30)	19 (15–25)	0.0133 *
**Complications, no. (%)**			0.0154 *
**Grade 1–2**	8 (10)	11 (14)	
**Grade ≥3**	13 (16)	2 (3)	
**Postoperative ileus, no. (%)**	11 (14)	4 (5)	0.0425 *
**Pathological T stage, no. (%)**			0.5516
pT0–1	20 (25)	24 (30)	
pT2–4	21 (26)	15 (19)	
**Adjuvant chemotherapy, no. (%)**	7 (9)	8 (6)	0.8374

ECUD, extracorporeal urinary diversion; ERAS, enhanced recovery after surgery; IC, ileal conduit; ICUD, intracorporeal urinary diversion; IQR, interquartile range; LOS, length of stay; NB, neobladder. * Indicate significant differences.

## Data Availability

The data presented in this study are available on request from the corresponding author due to the decision of the Ethics Committee.

## Abbreviations

The following abbreviations are used in this manuscript:

ERAS	Enhanced recovery after surgery
RARC	Robot-assisted radical cystectomy
LOS	Length of stay
GDFT	Goal-directed fluid therapy
ROC	Receiver operating characteristic
ECUD	Extracorporeal urinary diversion
ICUD	Intracorporeal urinary diversion
IC	Ileal conduit
NB	Neobladder
